# Supplementary material for: Short‐term insurance versus long‐term bet‐hedging strategies as adaptations to variable environments
Source: Evolution. 2018 Dec 26;73(2):145–57. doi: 10.1111/evo.13659 (PMC6590291; doi:10.1111/evo.13659)
Supplement: Supplementary file 1 — Appendix S1: R code [file EVO-73-145-s001.docx]

Supporting information to:

Short-term insurance versus long-term bet-hedging strategies as adaptations to variable environments

# Appendix S1: R code

################################################################

# Short-term insurance versus long-term bet hedging strategies #

######### as adaptations to variable environments ##########

################################################################

rm(list=ls())

library(emdbook)

library(RColorBrewer)

## Skew normal fitness function (eq. 1)

## Arguments:

## z: A phenotype or a vector of phenotypes, for which fitness is to be calculated

## theta: Location parameter. Defaults to 0.

## omega: Width parameter. Defaults to 1. var(w.sn)=omega^2 only when alpha=1.

## alpha: Skew parameter. Defaults to 0 (no skew).

w.sn <- function(z,theta=0,omega=1,alpha=0) {

2*dnorm(z,mean=theta,sd=omega)*pnorm((z-theta)/omega*alpha)

}

#Find function maxima (theta_0) for various alpha values

xs <- seq(-3,3,by=0.001)

alphas <- c(0,1,2,5,10,50) #seq(-5,5,by=0.5)

mode.w <- numeric(length(alphas))

for(i in alphas){

mode.w[which(alphas==i)] <- optimize(w.sn,range(xs),maximum=TRUE,alpha=i)$maximum

}

## Function to calculate variance of skew normal fitness function with a given omega and alpha.

## (Source: https://en.wikipedia.org/wiki/Skew_normal_distribution)

var.sn <- function(omega=1,alpha=0){

delta <- alpha/sqrt(1+alpha^2)

return(omega^2*(1-(2*delta^2)/pi))

}

## wbar: when there is phenotypic variation in the genotype.

## This is a generic wbar function for any w(k) (general form of eq. 2)

## Uses numerical integration so analytical form is better (as wbar.sn below).

##

## Arguments:

## mu: mu_k, mean phenotype expressed by genotype k

## sigma: sigma_k, standard deviation of phenotypes expressed by genotype k

## wfun: w(z), the fitness function

## log: Whether to return log fitness, or not (default)

## ...: Additional parameters to be passed to wfun (such as location or shape)

wbar <- function(mu, sigma, wfun, log=FALSE,...) {

if (sigma==0)

tmp <- wfun(mu,...)

else {

integrand <- function(z) {

wfun(z,...)*dnorm(z,mu,sigma)

}

tmp <- integrate(integrand,-Inf,Inf)$value

}

if (log)

log(tmp)

else

tmp

}

## wbar.sn: Analytically calculates wbar (eq. 2, final expression)

## Arguments as in w.sn and wbar.

wbar.sn <- function(mu,sigma,theta=0,omega=1,alpha=0,log=FALSE) {

if (log) {

tmp <- omega^2 + sigma^2

log(2) + log(omega) - .5*log(tmp)

- (mu-theta)^2/(2*tmp)

+ pnorm(omega*alpha/sqrt(omega^2 + sigma^2*(1+alpha^2))*(mu-theta)/sqrt(tmp),log.p=TRUE)

} else {

(omega^2+sigma^2)^(-.5)*w.sn(mu-theta,0,omega=sqrt(omega^2+sigma^2),alpha=alpha)

}

}

# Plot fig. 1C

par(mfrow=c(1,1),mar=c(6,6,1,1))

xs <- seq(-2,3,by=0.001)

cols <- brewer.pal(6,"Reds")

wbarMax <- list()

plot(xs,w.sn(xs,-mode.w[which(alphas==5)],1,5),type="l",lwd=2,

ylab=expression("Fitness ("*italic(w)*")"),xlab=expression("Phenotype ("*italic(z)*")"),cex.lab=1.3)

arrows(0,-0.03,0,w.sn(0,-mode.w[which(alphas==5)],1,5),lty=2,length=0)

sigmas <- c(2,1.5,1,0.5)

for(i in 1:length(sigmas)){

curve(wbar.sn(x,sigmas[i],theta=-mode.w[which(alphas==5)],1,5),add=TRUE,col=cols[i+2])

wbarMax[[i]] <- optimize(wbar.sn,range(xs),maximum=TRUE,theta=-mode.w[which(alphas==5)],omega=1,alpha=5,sigma=sigmas[i])

arrows(wbarMax[[i]]$maximum,-0.03,wbarMax[[i]]$maximum,wbarMax[[i]]$objective,lty=2,length=0,col=cols[i+4])

}

mtext(expression("Genotype fitness ("~italic(bar(w))*")"),2,2,col="Red")

mtext(expression("Mean phenotype ("~italic("\u03bc"[k])*")"),1,4,col="Red")

## mean.wbar: Compute the geometric or arithmetic mean or the variance

## of mean fitness of a given genotype with phenotypic mean mu and

## variance sigma^2 given fluctuation in some parameter of the individual

## fitness function between generations

##

## Arguments:

## mu: mu_k, phenotypic mean of the genotype

## sigma: sigma_k, phenotypic standard deviation of the genotype

## wfun: The name of the individual fitness function w (first argument must

## be the phenotype)

## wbarfun: An optional name of a function computing the the mean fitness

## of a genotype mu,sigma. Use this instead of wfun if an

## analytic formula for this is available to avoid double

## numerical integration

## fluctname: A character string giving the name of the wfun parameter that

## fluctuates between generations (defaults to "theta")

## wpar: List of other parameters to wfun (such as scale and shape

## parameters)

## dfun: Density function specifying the distribution of fluctuations in parname

## Defaults to "dnorm"

## fluctpar: A list containing the parameter passed as arguments to dfun specifying the

## magnitude etc. of fluctuations in fluctpar. Defaults to list(sd=1)

## type: "geometric", "arithmetic" or "variance"

##

## Value:

## Geometric mean fitness

mean.wbar <- function(mu, sigma,

wfun=NULL,

wbarfun=NULL,

fluctname="theta",

wpar=NULL,

dfun="dnorm", fluctpar=list(sd=1),

type="geometric")

{

wpar <- c(mu=unname(mu),sigma=unname(sigma),log=type=="geometric",wpar)

if (is.null(wbarfun)) { # if no wbar function is given

wbarfun <- wbar # then computing this using numerical integration

if (is.null(wfun))

stop("No function wbarfun computing mean genotypic fitness or wfun computing phenotypic fitness must be given")

wpar <- c(wpar,wfun=wfun) # and tell wbar to call the individual fitness function

}

integrand <- function(fpar,power=1) {

tmp <- numeric(length(fpar))

for (i in 1:length(fpar)) {

wpar[[fluctname]] <- fpar[i] # add theta (or another fluctuating parameter) to argument list

fluctpar$x <- fpar[i] # specify the value of theta where we want the prob. density

tmp[i] <- do.call(wbarfun,wpar)*do.call(dfun,fluctpar)

}

tmp[is.nan(tmp)|tmp==-Inf] <- 0

tmp

}

result <- integrate(integrand,-Inf,Inf)$value

switch(type,

geometric=exp(result),

arithmetic=result,

variance={

secondmoment <- integrate(integrand,-Inf,Inf,power=2)$value

secondmoment - result^2

})

}

## Creating 3d plots using curve3d from package emdbook.

## Arguments:

## alpha: skew parameter for fitness function, defaults to 0

## sdenv: sigma_theta; standard deviation of the normal distribution from which env. fluctuations are drawn

## xlim,ylim: Vectors of c(lower,upper) limits of x- and y-axis. Note that other settings than default may

## give errors with divergent integrals.

## arit: Whether to calculate arithmetic (default) or geometric (set arit=FALSE) mean fitness.

## xlab,ylab: Whether to print axis labels.

## main: Whether or not to print the sdenv parameter above the plots. (default no)

## ...: Additional parameters

curveplot <- function(alpha=0,sdenv=1,arit=TRUE,xlim=c(-5,5),ylim=c(0,10),xlab=FALSE,ylab=FALSE,main=FALSE,...){

a <<- alpha

mod <<- -mode.w[which(alphas==a)]

sdenv <<- sdenv

mn <- ifelse(main,sdenv,"")

xl <- ifelse(xlab,expression("Mean phenotype ("~mu[k]~")"),"")

yl <- ifelse(ylab,expression("Variance in phenotype ("~sigma[k]~")"),"")

if(sdenv==0){

yl <- expression("Variance in phenotype ("~sigma[k]~")")

curve3d(wbar(mu,sigma,w.sn,alpha=a,theta=mod),xlim=xlim,ylim=ylim,main=mn,

varnames=c("mu","sigma"),sys3d="contour",n=20,xlab=xl,ylab=yl,cex.lab=1.2)

}else{

if(arit){

curve3d(mean.wbar(mu,sigma,w.sn,fluctpar=list(sd=sdenv),wpar=list(alpha=a,theta=mod),type="arithmetic"),

xlim=xlim,ylim=ylim,varnames=c("mu","sigma"),main=mn,

sys3d="contour",n=20,xlab=xl,ylab=yl,cex.main=1.2)

} else{

curve3d(mean.wbar(mu,sigma,w.sn,fluctpar=list(sd=sdenv),wpar=list(alpha=a,theta=mod),type="geometric"),

xlim=xlim,ylim=ylim,varnames=c("mu","sigma"),main=mn,

sys3d="contour",n=20,xlab=xl,ylab=yl,cex.main=1.3,cex.lab=1.2)

}

}

}

##Example usage (plotting figure 3)

sdenvs <- c(0,0.5,1,1.5,2)

par(mfrow=c(2,length(sdenvs)),oma=c(4,4,2,0),mar=c(2,2.9,2,0.4))

for(i in sdenvs){ #The arithmetic mean fitness surfaces

curveplot(alpha=5,sdenv=i,main=TRUE)

}

for(i in sdenvs){ #The geometric mean fitness surfaces

curveplot(alpha=5,sdenv=i,arit=FALSE,main=FALSE)

}

mtext(expression("Standard deviation of phenotypic optimum, "*sigma[theta]),outer=TRUE,cex=1.3)

title(xlab=expression(" Mean phenotype ("*mu[italic(k)]*")"),cex.lab=1.6,line=1.7,outer=TRUE)

title(ylab=expression("Standard deviation of phenotype ("*sigma[italic(k)]*")"),cex.lab=1.6,line=0,outer=TRUE)

mtext("Geometric mean fitness Arithmetic mean fitness",side=2,cex=1.3,line=2.5,outer=TRUE)
